# Comparative evaluation of cannabinoid receptors, apelin and S100A6 protein in the heart of women of different age groups

**DOI:** 10.1186/s12872-018-0923-0

**Published:** 2018-10-04

**Authors:** Irena Kasacka, Żaneta Piotrowska, Anna Filipek, Wojciech Lebkowski

**Affiliations:** 1 Department of Histology and Cytophysiology, Medical University of Bialystok, Mickiewicza 2C street, 15-222 Białystok, Poland; 2 Nencki Institute of Experimental Biology, Laboratory of Calcium Binding Proteins, Ludwika Pasteura 3 street, 02-093 Warszawa, Poland; 3 Department of Neurosurgery, Medical University of Bialystok, Marii Skłodowskiej-Curie 24A street, 15-276 Białystok, Poland

**Keywords:** CB1, CB2, Apelin, S100A6, Women, Heart

## Abstract

**Background:**

Recent studies have shown a significant role of the endocannabinoid system, apelin and S100A6 protein in the regulation of cardiovascular system functioning. The aim of the study was to compare and evaluate the distribution of cannabinoid receptors (CB1 and CB2), apelin and S100A6 protein in the heart of healthy women in different age groups.

**Methods:**

The study was conducted on the hearts of 10 women (organ donors) without a history of cardiovascular disease, who were divided into two age groups: women older than 50 years and women under 50 years of age. Paraffin heart sections were processed by immunohistochemistry for detection of cannabinoids receptors (CB1 and CB2), apelin and S100A6 protein.

**Results:**

CB1 and CB2 immunoreactivity in the cytoplasm of cardiomyocytes in the heart of women over 50 was weaker than in younger individuals. There was also strong immunoreactivity of CB1 in intercalated discs (ICDs) of the heart, only in women over 50. The presence of this receptor in this location was not found in women under 50. Apelin- and S100A6-immunoreactivity in the cardiomyocytes was stronger in older women compared to women under 50.The CB1, apelin and S100A6 immunostaining in the endothelium of myocardial vessels was weaker in women over 50 than in younger women, while intensity of CB2- immunoreaction in coronary endothelium was similar in both groups of women.

The results of the study indicate the important role of endocannabinoids, apelin, and S100A6 protein in cardiac muscle function.

**Conclusion:**

This report might contribute to a better understanding of the role of endocannabinoid system, apelin and S100 proteins in heart function as well as shed new light on processes involved in age-related cardiomyopathy.

## Background

Since the discovery of the endocannabinoid system in the 1990’s, evidence indicating its key importance in various physiological and pathological processes within the body has been emerging [1]. The endocannabinoid system contributes to the control of mental health, eating behaviour, reproductive function, pain sensation and immune response [1]. Latest reports have also revealed a substantial role of the endocannabinoid system in cardiovascular system performance [1]. The cannabinoid receptors CB1 and CB2 and their endogenous ligands, called endocannabinoids, have been identified in cardiovascular tissues of human and several mammal species [1]. It has been proved that the endocannabinoid system participates in the regulation of blood pressure, heart rate and myocardial contractility [1, 2]. The endocannabinoid system also determines cardiomyocytes survivability and is involved in histopathological changes in the heart [3–8]. CB1 receptor axis promotes cardiomyocytes injury, augments collagen deposition and cardiomyocyte overgrowth in experimental models of cardiovascular diseases [3–5]. By contrast, treatment with CB2 receptor agonist limits cardiomyocytes apoptosis and prevents heart hypertrophy and fibrosis in rodents subjected to myocardial infarction (MI) and ischemia–reperfusion (I/R) heart injury [6–8].

Recent studies have shown the relevance of apelin in cardiovascular homeostasis [9]. Both the apelin receptor (APJ) and the apelin peptide have been detected in human and rat heart [9]. Clinical, experimental and in vitro studies have revealed that apelin lowers blood pressure, increases heart rate, evokes positive inotropic effect, and modulates cardiac loading [9, 10]. Literature data have also indicated the cardioprotective action of apelin. Treatment with apelin reduces myocardial injury, limits hypertrophy and fibrosis of cardiac tissue in rodents with MI, I/R heart injury and heart pressure overload [11–16]. Several reports have indicate the existence of a functional interaction between endocannabinoid system and apelin [17–19]. It was found that cannabinoid signalling modulates the expression of the apelin gene in the adipocyte and skeletal muscle cells [17–19]. In this way, it can be concluded that the endocannabinoid system influences the biological function of apelin, including its cardiovascular action.

It is known that the contractility of myocardial cells is strictly dependent on the fluctuation of intracellular calcium content. Cannabinoids and apelin modulate the calcium flow in cardiomyocytes by affecting calcium channels (L - type Ca^2+^ channels, T-type Ca^2+^ channels, Na+/Ca^2+^ exchanger) and calcium circulation between the sarcoplasmic reticulum and cytosol [1, 9, 10].

The calcium signalling in myocardial cells is regulated by various calcium-binding proteins. They also include proteins belonging to the S100 family that have two EF-hand type calcium-binding domains [20].

Recent studies on the S100 proteins family have revealed that its members, S100A6, fulfils an important role in maintaining heart functionality [21]. It has been demonstrated that S100A6 regulates calcium transition between the sarcoplasmic reticulum and the cytosol in cardiomyocytes [21]. This finding indicates the involvement of S100A6 in intracellular calcium cycling during cardiac muscle cells contraction. Moreover, it has been stated that S100A6 regulates cardiomyocytes differentiation and exerts a beneficial effect on cardiomyocytes viability [21, 22]. Studies on mice and cultured neonatal rat cardiomyocytes proved protective role of S100A6, limiting apoptosis of cardiac muscle cells [21, 22]. By contrast, in vitro studies on carcinoma cells lines have demonstrated that S100A6 might participate in programmed cell death pathways [23, 24].

Aging is a major risk factor for cardiovascular morbidity and mortality [25]. Clinical and experimental data have demonstrated that aging is accompanied by histopathological changes in the heart such as cardiomyocyte apoptosis, cardiac muscle cell hypertrophy and heart fibrosis [26]. Age-associated heart remodelling is followed by cardiac dysfunction. Haemodynamic studies have revealed that elderly patients have a slower heart rate, decreased cardiac output, impaired cardiac systolic and diastolic function compared to younger individuals [26].

Considering the above-mentioned unfavourable impact of aging on the cardiovascular system and the involvement of the cannabinoid system, apelin and S100A6 protein in control of cardiac function, we decided to perform immunohistochemical detection and comparative evaluation of the distribution of cannabinoid receptors (CB1 and CB2), apelin and S100A6 in the heart of healthy women in different age groups.

## Methods

### Sample collection

Ten adult women (organ donors) without history of cardiovascular disease were used in the study. The women were in age range from 19 to 64 yrs., mean body weight 63,2 kg and mean BMI (body mass index) 23,3 kg/m^2^.

The women were divided into two groups: subjects older than 50 years (over 50) and subjects under 50 years old (under 50).

Each study participant presented with clinical symptoms of brain death, was considered to be an organ donor. Irreversible brain damage was confirmed by special clinical examination and angiography (no blood flow within the brain arteries). After brain death was diagnosed and confirmed, heart samples were collected from each body after other organs (kidneys, liver) for transplantation were harvested.

Heart samples were immediately fixed in Bouin’s solution and routinely embedded in paraffin. Sections (4 μm) were stained with haematoxylin-eosin for general histological examination and processed by immunohistochemistry for detection of cannabinoids receptors (CB1 and CB2), apelin and S100A6 protein.

### Ethical issues

The study protocol was approved by the Ethics Committee, Medical University of Białystok (R-I-002/345/2007), and a written informed consent had previously been obtained from each woman or from her family member(s).

### Immunohistochemistry

Paraffin blocks were cut into 4-μm sections (3 sections from each subject for each antibody) and attached to positively charged glass slides. Immunohistochemistry was performed, using an EnVision Plus-HRP Rabbit Detection Kit K4011 (Dako Denmark) [27]. Immunostaining was performed by the following protocol: paraffin-embedded sections were deparaffinized and hydrated in pure alcohols. For antigen retrieval, the sections were subjected to pre-treatment in a pressure chamber heated for 1 min at 21 psi at 125 °C (one pound force per square inch (1 psi) equates to 6.895 kPa, the conversion factor has been provided by the United Kingdom National Physical Laboratory). During antigen retrieval sections were incubated with Target Retrieval Solution Citrate pH = 6.0 S 2369 (Dako Denmark) for CB2 and S100A6, or Target Retrieval Solution with pH 9.0 (S 2367, Dako Denmark) for CB1 and apelin. After cooling down to room temperature, the sections were incubated with Peroxidase Blocking Reagent S 2001 (Dako, Denmark) for 10 min to block endogenous peroxidase activity. Subsequently, the sections were incubated overnight with the primary antibodies against CB1 (No ab23703 purchased from Abcam, UK), CB2 (No ab3561 purchased from Abcam, UK) S100A6 (purchased from Nencki Institute of Experimental Biology, produced in-house) and apelin (No. bs-2425R purchased from Bioss Antibodies) at 4 °C in a humidified chamber. The antisera were previously diluted in Antibody Diluent (S 0809, Dako Denmark), in proportion 1:1000 for CB1, 1:200 for CB2 and apelin, 1:5000 for S100A6. The procedure was followed by incubation with secondary antibody (conjugated to horseradish peroxidase-labelled polymer). The bound antibodies were visualized by 1 min incubation with liquid 3,3^′^-diaminobenzidine (DAB) substrate chromogen. The sections were finally counterstained in QS haematoxylin (H-3404, Vector Laboratories, Burlingame, CA, USA), mounted and evaluated under light microscope. Appropriate washing with S 3006 Wash Buffer (Dako Denmark) was performed between each step. Specificity tests, performed for the CB1, CB2, apelin and S100A6 antibody included: negative control, where the antibodies were replaced by normal rabbit serum (Vector Laboratories, Burlingame, CA, USA) at respective dilution. For negative control, no immunostaining was observed in heart tissues under the omission of the primary antibodies.

Histological preparations were subjected to a visual analysis using an Olympus BX41 light microscope with Olympus DP12 digital camera and a PC computer and documented.

### Quantitative analysis

Images from five randomly selected microscopic fields, each field of 0.785 mm^2^, in magnification of 200× (20× the lens and 10× the eyepiece) from all heart sections were submitted for morphometric evaluation by using NIS Elements AR 3.10 Nikon software for microscopic image analysis.

The intensity of immunohistochemical reaction was measured, using a 0 to 256 grey scale level, where the completely **white or bright pixels** were scored **0** and completely **black** pixels were scored **256**.

### Statistical analysis

All data were analysed for statistical significance using software computer package Statistica Version 12.0. The mean values were computed automatically; significant differences were determined by Student’s t-test; *p* < 0.05 was accepted as significant**.**

## Results

Routine tests (H + E staining) showed no microscopic pathological changes in the hearts of women.

The mean values of age, body weight and body mass index (BMI) in the two groups of women are presented in Table 1. The average body weight and BMI of women in our study was similar.

**Table 1:** Age (year), weight (kg) and BMI (body mass index - kg/m^2^) of women expressed as mean ± SE

Group of women	age (years)	weight (kg)	BMI (kg/m^2^)
women under 50 years old			
*n* = 5	31.4 ± 4.36	61.9 ± 2.21	22.6 ± 0.93
women over 50 years old			
*n* = 5	57.0 ± 2.04 *	66.6 ± 4.44	24.5 ± 2.17

*p < 0.05 women over 50 vs women under 50

**Fig. 1:**
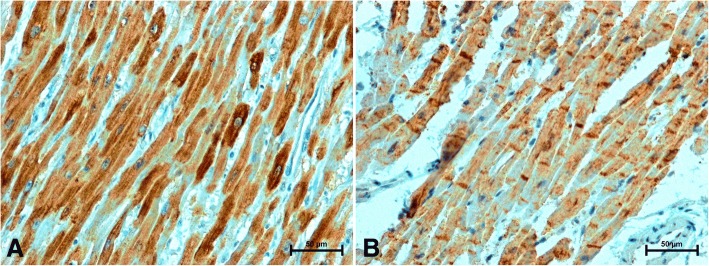
Immunolocalization of CB1 receptor in the heart of woman (**a**) under 50 years old; strong CB1-immunosignal in cardiomyocytes (**b**) over 50 years old; weak CB1 immunoreactivity in the cytoplasm of cardiomyocytes and very strong CB1-immunolabelling in ICDs

**Fig. 2:**
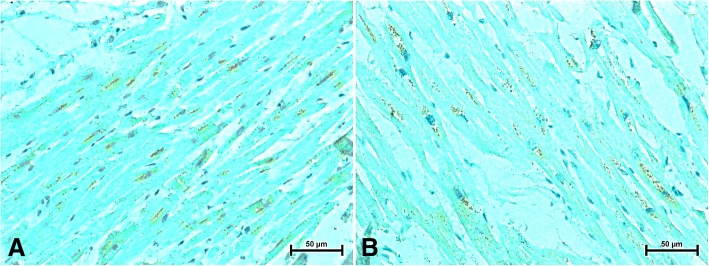
Immunodetection of CB2 receptor in the heart of women (**a**) under 50 and (**b**) over 50 years old

**Fig. 3:**
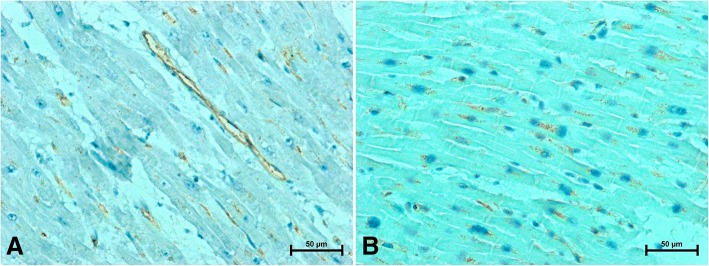
Representative images of apelin immunolabeling in the heart of women (**a**) under 50 (**b**) over 50 years old

**Fig. 4:**
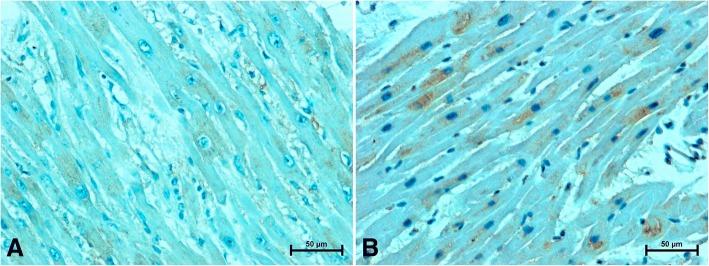
Positive S100A6-immunostaining in heart of women (**a**) under 50 years old and (**b**) women over 50

The performed immunohistochemical tests revealed a positive reaction of CB1, CB2 receptors, apelin and S100A6 in the heart of all studied women, although the density and intensity of reactions varied between age groups (Figs. 1, 2, 3 and 4). There was no immunoreactivity when the primary antibodies were omitted from the staining procedure.

Immunolabelling of CB1 in the hearts of women under 50 gave a moderate to strong reaction in the cytoplasm of cardiomyocytes (Fig. 1a). In women over 50, the intensity of CB1 immunoreactivity in the cytoplasm of myocardial cells was significantly weaker, but in the hearts of these women strong reactivity of the CB1 receptors in the intercalated discs was observed (ICDs) (Fig. 1b). In the heart of women under 50 was noted also weak CB1-immunostaining in endothelium of coronary vasculature (Fig. 1a), in vessels supplying the heart of older women the CB1-immunoreaction was residual or not detected (Fig. 1b).

The same perinuclear CB2 receptor in cardiomyocytes was found in the hearts of women in both groups (Fig. 2a and b). The intensity of CB2-immunoreaction in cardiac muscle cells was weaker in the hearts of older women compared to those under 50 (Fig. 2b). In all studied women was observed similar, very weak CB2-staining in endothelium of myocardial vessels (Fig. 2a and b).

Antisera against apelin strongly immunostained vascular endothelial cells and gave a very weak reaction in the cardiomyocytes in the hearts of younger women (Fig. 3a). In the hearts of women older than 50 years, the apelin immunosignal in endothelial cells was very weak or negative (Fig. 3b), whereas the density and intensity of the reaction showing apelin in the cardiomyocytes of women over the age of 50 was far stronger than in younger women (Fig. 3a and b).

In the hearts of younger women a weak S100A6-immunoreactivity in the cytoplasm of cardiac muscle cells was noted. The S100A6-immunosignal was observed in the form of brown-stained granules in the vicinity of cardiomyocyte nuclei (Fig. 4a). The S100A6-immunoreaction in the cardiomyocytes of older women was considerably stronger compared to subjects under 50 (Fig. 4b). The S100A6 was identified also in endothelial cells of coronary vasculature. In the hearts of women under 50, the S100A6-immunostaining in endothelial cells was moderate (Fig. 4a), while in the hearts of women over 50 weakened S100A6-immunoreaction in vascular endothelium was noted (Fig. 4b).

Computer image analysis confirmed the visually perceived changes in the intensity of the immunohistochemical reaction against CB1, CB2, apelin and S100A6 in the hearts of women over 50 and under 50 years of age (Table 2).

**Table 2:** Intensity of immunoreaction against CB1, CB2, apelin and S100A6 in women heart (mean ± SE). (Scale from 0 (white pixel) to 256 (black pixel))

Intensity of immunohistochemical reaction in women heart	women under 50 years old	women over 50 years old	
CB1			
cardiomyocyte cytoplasm	146.7 ± 2.99	108.2 ± 2.45*	↓*
intercalated discs	ND	180.7 ± 4.63*	↑*
endothelium of coronary vessels	61.6 ± 2.16	45.7 ± 2.43*	↓*
CB2			
cardiomyocyte cytoplasm	110.7 ± 2.57	91.1 ± 3.57*	↓*
endothelium of coronary vessels	49.9 ± 2.15	48.1 ± 1.98	
apelin			
cardiomyocyte cytoplasm	73.6 ± 2.26	95.0 ± 2.72*	↑*
endothelium of coronary vessels	135.9 ± 3.65	73.9 ± 2.43*	↓*
S100A6			
cardiomyocyte cytoplasm	81.0 ± 1.85	125.9 ± 3.30*	↑*
endothelium of coronary vessels	97.4 ± 3.84	79.1 ± 2.48*	↓*

*p < 0.05 women over 50 vs women under 50

↓ weakening of immunohistochemical reaction

↑ intensification of immunohistochemical reaction

*ND* not detected

## Discussion

The cardiovascular system is subject to precise regulation in order to ensure appropriate blood supply to different body tissues under a wide range of circumstances. The results of the latest research demonstrate that the endocannabinoid system, apelin and S100A6 participate in the complex process controlling the cardiovascular system function since they exert a significant influence on blood pressure, heart rate and myocardial contractility [1, 9, 21].

To the best of our knowledge, the current report is the first comparison of CB1, CB2, apelin and S100A6 distribution in the hearts of healthy women of different age groups. Our study demonstrated decreased immunoreactivity for both cannabinoid receptors, while increased immunohistochemical reaction for apelin and S100A6 in the cardiomyocyte cytoplasm in the hearts of older women. Noteworthy is the fact that in women over 50 the presence of CB1 receptor was detected also in ICDs, which was not the case in younger women. Presented research indicate also on decrease of CB1, apelin and S100A6 immunoreactivity, but no significant changes in intensity of CB2 immunostaining in the endothelium of coronary vessels in women over 50.

Aging is associated with substantial alterations in cardiovascular structure and function. Aging-related vascular stiffness and blood pressure elevation leads to considerable cardiac workload. The increasing pressure acting on the heart’s wall results in myocardial hypertrophy and fibrotic remodelling of the cardiac wall in older individuals [26, 28]. Moreover, aging leads to the activation of multiple molecular mechanisms resulting in cardiomyocyte damage. These include a decrease in the efficiency of somatic mutations repair, the accumulation of defective proteins as well as increased oxidative stress due to the impairment of mitochondrial bioenergetics [28].

The growing body of evidence points to the involvement of the endocannabinoid system, apelin-APJ system and S100A6 protein in histopathological changes in the heart [1, 9, 21]. The activation of CB1 and CB2 receptors exerts a significant impact on cardiomyocyte survival. Recent investigations on mice models of cardiomyopathy have demonstrated that CB1-signalling contributes to programmed cell death while the CB2-axis produces a cardioprotective effect [3–8]. In vivo and in vitro studies have revealed that cardiac muscle cell viability is also dependent on apelin. Apelin prevented cardiomyocyte death in cases of I/R heart injury or glucose deprivation [11–13]. A few previous studies have demonstrated the importance of S100A6 in cardiomyocyte survival. Mofid et al. [21] and Tsoporis et al. [22] demonstrated that S100A6 markedly reduced the apoptosis of cardiomyocytes exposed to TNF-α and hypoxia/reoxygenation conditions. By contrast, other researchers have stated that S100A6 promotes apoptosis in different cultured cell types [23, 24].

Experimental data indicate that the endocannabinoid system participates in the process of cardiac fibrosis. It has been noted that the CB1 receptor-axis determines collagen deposition in the heart of rodents subjected to MI, doxorubicin-induced myocardial injury and diabetic cardiomyopathy [3–5]. In turn, the stimulation of CB2 receptor decreased fibrotic remodelling of the heart’s wall in mice undergoing an I/R episode and MI [7, 8]. Some recent evidence highlights the antifibrotic action of apelin in cardiovascular disease. Apelin has been found to restrict cardiac fibrosis in rodents subjected to heart pressure overload, Dahl-salt hypertension, pulmonary hypertension or MI [14, 15, 29]. Recent investigations indicate that S100A6 protein attenuates the pathological fibrotic remodelling of the cardiac wall. It has been demonstrated that mice overexpressing S100A6 have less collagen-rich scar content in the heart post I/R injury compared to animals in which S100A6 gene was not influenced [21].

The endocannabinoid system performs a relevant role in the progression of heart hypertrophy. Studies on mice with experimental MI and diabetes have demonstrated that the CB1-pathway is implicated in cardiomyocyte overgrowth, whereas CB2-signalling is associated with protection against cardiac hypertrophy [4, 5, 8]. Several recent reports have demonstrated that apelin limits the development of heart hypertrophy. Apelin treatment substantially reduced cardiac hypertrophy in rodents with heart pressure overload and various models of hypertension [14, 15, 30]. In the past decade researches have confirmed the involvement of S100A6 protein in cardiac hypertrophy. It has been stated that S100A6 abolishes cardiomyocyte overgrowth induced by various hypertrophic factors and reduces cardiac hypertrophy in mice exposed to I/R heart injury [21, 31].

In view of the aforementioned, the changes in CB1, CB2, apelin and S100A6 levels in the hearts of women over 50 observed in our study might suggest the involvement of the endocannabinoid system, apelin and S100A6 protein in age-related heart remodelling. However, as the current study provides novel findings, further research should be performed to thoroughly understand the importance of CB1, CB2, apelin and S100A6 in age-induced cardiovascular changes.

It is known that in patients above the age of 50–60, dysfunction of the mechanical action of the heart occurs [26]. There are overwhelming literature data proving the impact of the endocannabinoid system on the frequency and strength of cardiac contraction. The activation of CB1 receptors evokes bradycardia and negative inotropic effect [1, 2]. In contrast to CB1 receptor, the stimulation of CB2-receptor triggers a positive contractile response in cardiac muscle cells [1, 2]. The systolic and diastolic heart function is also regulated by the apelin-APJ system [10]. Apelin treatment has significantly increased cardiomyocyte contractility in physiological and pathological conditions [10, 14, 15, 29]. A few reports have indicated that S100A6 might influence cardiac contractility. It has been demonstrated that S100A6 regulates calcium cycling between the sarcoplasmic reticulum and the cytosol in cardiomyocytes, which is necessary for the occurrence of the contraction cycle [21].

On the basis of this, a possible link might be suspected between impaired cardiac performance in older individuals and the alterations in the cannabinoid system, apelin and S100A6 in the hearts of women over 50 demonstrated in the present study.

Clinical and experimental studies have demonstrated abnormal activity of the endocannabinoid system in cardiovascular diseases including myocardial infarction, I/R heart injury, obesity-related circulatory dysfunction, diabetic cardiomyopathy and doxorubicin-induced heart failure [1, 3, 6, 8]. Numerous literature reports have indicated that cardiovascular diseases progress together with the impairment of apelin expression. Reduced circulating apelin levels have been observed in patients with heart failure, coronary artery disease and lone atrial fibrillation as well as in experimental models of isoproterenol-induced cardiomyopathy, I/R heart injury and heart failure [11, 32–36]. Cardiovascular events have also been linked with S100A6 upregulation. Increased S100A6 serum levels have been found in patients with acute coronary syndrome and in the rat model of MI [37].

Considering the above, the alterations in CB1, CB2, apelin and S100A6 distribution in the hearts of older women might be related with the development of cardiovascular complications in older individuals.

Conducted immunohistochemical identification of cannabinoid receptors in heart of women gave positive reaction for CB1 and CB2 receptors in cytoplasm of cardiac cells. The presented observation is consistent with results of Weis et al. [38], who similarly stated immunoreactivity for cannabinoid receptors in cardiomyocytes cytoplasm in human heart. Cannabinoid receptors belong to G-protein coupled membrane receptor family [1].

The G-protein coupled receptors are localized not only on cell membrane, they have been identified also on intracellular organelles including mitochondria [39]. Mendizabal-Zubiaga et al. [40] investigated distribution of cannabinoid receptors in cardiac muscle cells using immunogold staining method and electron microscope. Authors revealed that cannabinoid receptors are disposed on cardiomyocytes sarcolemma and also on the membrane of intracellular organelles. The greatest density of immunoparticles showing cannabinoid receptors was observed on mitochondrial membrane [40]. Described in our report cytoplasmic immunostaining for cannabinoid receptors in cardiomyocytes might be explained by the attachment of antibodies against CB1 and CB2 to receptors located on organelles membrane.

In the current study we observed the appearance of a strong CB1-immunosignal in ICDs in the hearts of women over 50, while in younger women CB1 was not detectable in this location. Intercalated discs ensure communication between cardiomyocytes in order to coordinate their function. ICDs construction contains adherens junctions, desmosomes and gap junctions and also a number of ion channels enabling the simultaneous spread of electrical potential across cardiac muscle cells. Voltage-dependent Ca^2+^ and Na^+^ channels are also among ion channels identified in ICDs [41]. Experimental studies have demonstrated that CB1 signalling modulates the activity of calcium and sodium voltage-gated channels [1]. Perhaps, the observed displacement of CB1 receptor to intercalated discs in the hearts of women over 50 is associated with the role of CB1 receptors in the regulation of ion current through channels occurring in ICDs.

Aging proceeds with progressive endothelial dysfunction, involving impaired secretion of vasoactive, proinflammatory and prothrombotic factors by endothelial cells as well as endothelial cell injury induced by intensified production of reactive oxygen species [42].

Enlarging literature data indicate on significant effect of cannabinoids, apelin and S100A6 on endothelial cell functioning. CB1-signaling impairs endothelium-dependent vascular dilatation and promotes endothelial cells apoptosis via inducing oxidative stress in those cells [43]. Whereas the CB2-pathway evokes endothelial-protective effect by attenuating endothelial cell response to proinflammatory molecules and limiting adhesion of monocytes to vascular endothelium [43]. Apelin triggers the endothelial release of vasodilator substances such as nitric oxide, therefore is involved in endothelial-dependent regulation of vascular tone [44]. The S100A6 protein regulates proliferative potential of endothelial cells and increases viability of those cells [45].

In the presented study we observed decrease of CB1 immunoreaction while unaltered immunoreactivity for CB2 in endothelium of coronary vasculature in older women. Having regard to above-mentioned, our findings might suggest inhibition of CB1-pathway in favour of CB2-signaling as adaptive process limiting endothelial dysfunction during ageing. However, this aspect should be subjected to further detailed investigation.

The current research revealed also weakened apelin and S100A6 immunostaining in endothelium of myocardial vessels in women over 50, which might indicate on participation of apelin and S100A6 in age-related deterioration of endothelial functioning.

## Conclusion

The presented results of immunohistochemical study demonstrate alterations in the cannabinoid system, apelin and S100A6 in the hearts of women over 50.

The observed changes in CB1, CB2, apelin and S100A6 in heart of older women might be the result of ageing induced dysregulation of heart homeostasis or an adaptive mechanism attenuating the development of cardiac complications in older individuals.

The role of endocannabinoid system, apelin and S100 protein in regulating the heart’s function during ageing and their importance in age-related cardiomyopathy require further investigation.

## Abbreviations

APJ: The apelin receptor
CB1: The cannabinoid receptor type 1
CB2: The cannabinoid receptor type 2
I/R: Ischemia/reperfusion
ICDs: Intercalated discs
MI: Myocardial infarction
TNF-α: Tumour necrosis factor-alpha
